# Oral Metallo-Beta-Lactamase Protects the Gut Microbiome From Carbapenem-Mediated Damage and Reduces Propagation of Antibiotic Resistance in Pigs

**DOI:** 10.3389/fmicb.2019.00101

**Published:** 2019-02-05

**Authors:** Sheila Connelly, Brian Fanelli, Nur A. Hasan, Rita R. Colwell, Michael Kaleko

**Affiliations:** ^1^Synthetic Biologics, Inc., Rockville, MD, United States; ^2^CosmosID, Inc., Rockville, MD, United States; ^3^University of Maryland Institute for Advanced Computer Studies, College Park, MD, United States

**Keywords:** beta-lactamase, antibiotic resistance, gut microbiome, dysbiosis, porcine (pig) model

## Abstract

Antibiotics can damage the gut microbiome, leading to serious adventitious infections and emergence of antibiotic resistant pathogens. Antibiotic inactivation in the GI tract represents a strategy to protect colonic microbiota integrity and reduce antibiotic resistance. Clinical utility of this approach was established when SYN-004 (ribaxamase), an orally-administered beta-lactamase, was demonstrated to degrade ceftriaxone in the GI tract and preserve the gut microbiome. Ribaxamase degrades penicillins and cephalosporin beta-lactams, but not carbapenems. To expand this prophylactic approach to include all classes of beta-lactam antibiotics, a novel carbapenemase, formulated for oral administration, SYN-006, was evaluated in a porcine model of antibiotic-mediated gut dysbiosis. Pigs (20 kg, *n* = 16) were treated with the carbapenem, ertapenem (ERT), (IV, 30 mg/kg, SID) for 4 days and a cohort (*n* = 8) also received SYN-006 (PO, 50 mg, QID), beginning the day before antibiotic administration. ERT serum levels were not statistically different in ERT and ERT + SYN-006 groups, indicating that SYN-006 did not alter systemic antibiotic levels. Microbiomes were evaluated using whole genome shotgun metagenomics analyses of fecal DNA collected prior to and after antibiotic treatment. ERT caused significant changes to the gut microbiome that were mitigated in the presence of SYN-006. In addition, SYN-006 attenuated emergence of antibiotic resistance, including encoded beta-lactamases and genes conferring resistance to a broad range of antibiotics such as aminoglycosides and macrolides. SYN-006 has the potential to become the first therapy designed to protect the gut microbiome from all classes of beta-lactam antibiotics and reduce emergence of carbapenem-resistant pathogens.

## Introduction

The gut microbiota have coevolved with their mammalian hosts to form a complex ecosystem that plays a key role in health and disease (Ley et al., 2008). Antibiotics disrupt this bionetwork, causing dysbiosis, which is a perturbation of the normal microbial balance, and compromise colonization resistance rendering the host susceptible to opportunistic pathogens such as *Clostridium difficile* (Stevens et al., 2011; Crowther and Wilcox, 2015). Dysbiosis is associated with a diverse array of disorders including cardiovascular, inflammatory, metabolic, neurologic, and respiratory diseases (Lloyd-Price et al., 2016). In addition, dysbiosis promotes pathogen evolution by facilitating transfer of antibiotic resistance and virulence genes (Stecher et al., 2013), with the gut microbiome functioning as a reservoir of antibiotic resistance (Penders et al., 2013). Antibiotic-mediated microbiome damage is indefinite and cumulative, as microbiota alterations can persist for months or years after antibiotic exposure, with the risk of adventitious infection increasing with each administration cycle (Jernberg et al., 2007; Dethlefsen and Relman, 2011; Stevens et al., 2011; Blaser, 2016).

A strategy to protect the gut microbiome from antibiotic collateral damage is to limit exposure of the colonic microbiota to antibiotics without compromising infection control efficacy. Animal and human studies with SYN-004 (ribaxamase), an orally-administered beta-lactamase enzyme intended for use with intravenous (IV) beta-lactams, verified that degrading antibiotics in the upper GI tract protected the gut microbiome from antibiotic damage and reduced emergence of antimicrobial resistance (Kaleko et al., 2016; Kokai-Kun et al., 2016; Connelly et al., 2017; Kokai-Kun J. et al., 2017; Kokai-Kun J. F. et al., 2017). Further examination of this prevention approach in a phase 2b clinical study verified its viability as ribaxamase was demonstrated to significantly reduce *Clostridium difficile* infection (CDI) in high-risk patients who were receiving ceftriaxone for treatment of a lower respiratory tract infection (Kokai-Kun J. et al., 2017; Clinicaltrials.gov, 2018). Notably, this approach protected the gut microbiome from antibiotic damage and limited emergence of antimicrobial resistance (Kokai-Kun J. et al., 2017).

Ribaxamase efficiently degrades beta-lactam antibiotics, including penicillins and most cephalosporins (Kaleko et al., 2016), but it does not inactivate carbapenems. Beta-lactams represent the most widely used class of broad-spectrum antimicrobials (Ozdalga, 2011) and were the only drug significantly associated with gut microbiome disruption in a comprehensive phenotype-controlled microbiome variation analysis of over 3900 participants (Falony et al., 2016). Of the beta-lactams, carbapenems are especially damaging. Carbapenems were ranked highest risk for resistance emergence in combination with activity spectrum (Weiss et al., 2015), and when compared directly to amoxicillin in porcine gut dysbiosis models, the carbapenem, ertapenem (ERT), affected greater microbiome disruption (Connelly et al., 2018). Carbapenems are considered a last resort compound prohibited for use in food animals and prescribed judiciously in humans (EFSA, 2013). However, despite these policies, the use of carbapenems (Klein et al., 2018) and the number of resistant infections (Johnson and Woodford, 2013) continue to climb worldwide. Indeed, the Center for Disease Control have declared carbapenem-resistant Enterobacteriaceae (CRE) an “urgent threat” (Centers for Disease Control and Prevention, 2013), which is exemplified by the finding that CRE infection is associated with high mortality (Martin et al., 2018). Furthermore, carbapenem use poses a strong risk for development of CDI (Vardakas et al., 2016; Watson et al., 2018), responsible for 29,000 annual deaths in the United States (Magill et al., 2014; Lessa et al., 2015). Therefore, protection of the gut microbiome from broad-spectrum beta-lactam antibiotics, including carbapenems, is predicted to diminish antibiotic collateral damage, reduce opportunistic pathogen infections, and mitigate emergence of antimicrobial resistance.

To expand microbiome protection to all classes of beta-lactams, a novel metallo-beta-lactamase, P2A, isolated from *Bacillus cereus* (previously named targeted recombinant beta-lactamase 2) (Stiefel et al., 2005), was characterized. P2A demonstrated inactivation of a broad spectrum of beta-lactams including penicillins, cephalosporins, carbapenems, as well as antibiotic/beta-lactamase inhibitor combinations *in vitro* (Stiefel et al., 2005; Connelly et al., 2019). Further *in vitro* characterization revealed that P2A lost function at low pH (≤5.5), while retaining biological activity in the presence of human intestinal fluid, a key requirement for an enzyme intended for function in the GI tract (Connelly et al., 2019). Despite pH sensitivity, orally-administered P2A was demonstrated to preserve colonization resistance in mice treated with the antibiotic-beta-lactamase inhibitor combination, piperacillin-tazobactam (Stiefel et al., 2005). Therefore, to optimize enzyme function *in vivo*, P2A was formulated for oral delivery similarly to ribaxamase (Bristol et al., 2017) by use of an enteric coating intended to protect the enzyme from high acid conditions of the stomach, with enzyme dissolution occurring in the upper small intestine, at pH ≥ 5.5 (Connelly et al., 2019). Formulated P2A was named SYN-006 (Connelly et al., 2019).

In this work, an established porcine model of antibiotic-mediated gut dysbiosis caused by IV administration of the carbapenem, ERT, was employed (Connelly et al., 2018). An analogous porcine model was used previously to verify ribaxamase efficacy for gut microbiome protection in animals treated with IV ceftriaxone (Connelly et al., 2017). Here the ability of SYN-006 to attenuate ERT-mediated gut microbiome damage and reduced emergence of antibiotic resistance was evaluated.

## Materials and Methods

### Test Article

SYN-006 is the oral formulation of P2A (targeted recombinant beta-lactamase 2), a Class B1 metallo-beta-lactamase isolated from *B. cereus* (Stiefel et al., 2005). P2A was manufactured in *E. coli* and formulated for oral delivery by incorporation into Eudragit^®^-coated sucrose pellets designed for release of active enzyme at pH 5.5 or greater (Connelly et al., 2019). Formulation of P2A was similar to that of ribaxamase (Bristol et al., 2017). However, a hydroxypropyl cellulose isolation layer was included between the enzyme and enteric coating to protect the enzyme from exposure to the low pH Eudragit solution during pellet production (Connelly et al., 2019). Hard capsules suitable for oral delivery were filled with the pellets for total P2A content of 50 mg/capsule.

### Animals and Test Article Administration

Sixteen healthy 2 month old pigs, *Sus scrofa domestica*, Yorkshire cross, approximately 20 kg, were obtained from Archer Farms, Inc. (Darlington, MD, United States). After arrival to the test site, Noble Life Sciences, Inc. (Sykesville, MD, United States), the animals were quarantined/acclimated for 23 days during which time the health status of each animal was evaluated daily. Animals were fed Southern States Non-medicated Hog Feed (SSC-25-629001, Lot G6148) and, therefore, were never exposed to in-feed antibiotics. At the end of quarantine, all animals were deemed healthy and randomly divided into two groups. Group 1 (*n* = 8) received ertapenem (ERT), 30 mg/kg, IV, SID for 4 consecutive days and Group 2 (*n* = 8) received ERT plus SYN-006, 50 mg, PO, QID for 21 doses starting the day before ERT administration.

Animals were fed three times a day at 7:00 am, 12:00 pm, and 5:00 pm. Pigs received feed + apple juice beginning on study day -7 and throughout the dosing period. On study day 0, animals in Group 2 received one capsule of SYN-006 followed by a syringe of apple juice (pH 3.0), immediately prior to each feeding. Animals received their fourth SYN-006 dose at 10:00 pm with apple juice. Animals in Group 1 received a syringe of apple juice without SYN-006 following the same dosing schedule as Group 2. Animals had free access to water at all times. Apple juice was administered as described as a precaution to ensure that pig stomach pH remained below pH 5.5 to prevent dissolution of the Eudragit^®^-coated enzyme pellets and premature P2A enzyme release and inactivation prior to reaching the upper small intestine (Connelly et al., 2019).

Ertapenem was supplied as a powder (1 g/vial; Merck, NDC 0006-3843-71) and each vial was reconstituted with 10 mL of sterile water to create 100 mg/mL of injectable suspension. Ertapenem was administered through an IV catheter to animals under sedation. For sedation, a TELAZOL cocktail consisting of TELAZOL (50 mg/ml), ketamine (250 mg/ml) and xylazine (250 mg/ml) was administered intramuscularly at a dose of 0.5–1.0 mL/50 lbs to induce and maintain sedation. Once sedated, each pig received 0.3 mL/kg for a total of approximately 600 mg of ertapenem administered slowly through the IV catheter, followed by a heparinized saline flush. Ertapenem was delivered at the same time daily.

Blood was collected on Day 3 of antibiotic treatment. Blood was collected aseptically from the cranial vena cava of anesthetized animals at 1, 2, and 3 h after antibiotic administration. Each time, approximately 9 mL of blood was collected and dispensed into a serum separator vacutainer tube. After coagulation, samples were centrifuged and the serum transferred to a cryovial and stored at -80°C until shipment to the analysis laboratory. Fecal samples were collected twice, prior to initiation of the dosing (study day -1) and after the 7 am feeding and the final SYN-006 dose for Group 2 was administered, on study day 5. Fresh samples were collected at the time of defecation and placed directly into the OMNIgene^®^ GUT sample kit collection tubes (DNA Genotek, Ottawa, ON, Canada). Fecal samples were stored at room temperature until shipped at study end for analysis.

All animal procedures were conducted in accordance with principles and guidelines established by the Noble Life Sciences Institutional Animal Care and Use Committee in accordance with the Animal Welfare Act at Noble Life Sciences, Inc., (Sykesville, MD, United States). The protocol was approved by the Noble Life Sciences Institutional Animal Care and Use Committee. Noble Life Sciences, Inc., is fully accredited by the Association for Assessment and Accreditation of Laboratory Animal Care (AAALAC), has Office of Laboratory Animal (OLAW) assurance, and is USDA licensed.

### Ertapenem Serum Measurement

Serum was analyzed for ertapenem using a liquid chromatography turbo ion spray tandem mass spectrometry method (LC/MS/MS) following protein precipitation extraction using Applied Biosystems Triple Quad API 5000 and 5500 LC/MS/MS system with Turbo ion spray interfaces. The positive ions were measured in MRM mode. Ertapenem-d_4_ was used as an internal standard. The data were acquired and analyzed by Applied Biosystems “Analyst” software, versions 1.5 and 1.5.2. The lower limit of quantitation (LLOQ) was 0.205 μg/mL and the upper limit of quantitation (ULOQ) was 100.069 μg/mL. A calibration curve composed of blanks, two zero standards, and ten nonzero calibration standards covering a range of 0.205–100.069 μg/mL were analyzed with the samples. Quality control samples at three different concentration levels corresponding to 0.640, 40.028, and 80.055 μg/mL were analyzed with the samples. Analyte to internal standard peak area ratio values were used to construct the calibration curve and to determine sample concentrations. Linear regression with 1/x^2^ weighting was used to obtain the best fit of the data for the calibration curve. Ertapenem serum analysis assay was developed, validated, and performed by Sannova Analytical, Inc. (Somerset, NJ, United States). Statistical analyses were performed using Microsoft Excel 2016. *T*-test analyses were two-tailed with unequal variance.

### Fecal DNA Extraction, Whole Genome Shotgun Sequencing and Metagenomic Analyses

Total DNA was isolated from fecal specimens, using the MOBIO Power-Soil^®^ DNA Isolation Kit (Qiagen, Germantown, MD, United States), following manufacturer’s instructions. Each DNA sample was normalized in 3–18 μL of nuclease-free water to a final concentration of 0.5 ng μL^-1^, using the Biomek FX liquid handler (Beckman Coulter Life Sciences, Brea, CA, United States). Libraries were constructed using the Nextera XT Library Prep Kit (Illumina, San Diego, CA, United States). For each sample, an input of 0.5 ng was used in the tagmentation reaction, followed by 13 cycles of PCR amplification using Nextera i7 and i5 index primers and 2X KAPA master mix per the modified Nextera XT protocol. PCR products were purified using 1.0X speed beads and eluted in 15 μL of nuclease-free water. The final libraries were quantified by PicoGreen fluorometric assay (100X final dilution) and the concentrations were in the range of 0.1–4.0 ng μL^-1^. The libraries were pooled by adding an equimolar ratio of each, based on concentration determined by PicoGreen, and loaded onto a high sensitivity (HS) chip run on the Caliper LabChipGX (Perkin Elmer, Waltham, MA, United States). The base pair size reported was in the range of 301–680 bp. Samples were sequenced using a single Illumina HiSeq v3 flowcell by multiplexing eight libraries per lane targeting 25 million 100 bp reads per sample. Standard read quality assessments were performed prior to metagenomics analyses using open source BBDuk software from BBTools^1^ and all samples conformed to an average read quality of Q20 indicating 99% sequencing accuracy^2^. Reads per sample were consistent (60,000,000 ± 6,000,000 reads/sample), indicating equal read depth making subsampling unnecessary to ensure uniform population diversity.

Unassembled whole genome shotgun metagenomic sequencing reads were directly analyzed using the CosmosID, Inc., bioinformatics software package (CosmosID Inc., Rockville, MD, United States), as described (Hasan et al., 2014; Ponnusamy et al., 2016; Hourigan et al., 2018; Roy et al., 2018), to achieve bacterial identification to species, subspecies, and/or strain level and quantification of microorganism relative abundance. Briefly, the system utilizes a high performance data-mining k-mer algorithm and highly curated dynamic comparator databases (GenBook^®^) that rapidly disambiguate millions of short reads into the discrete genomes or genes engendering the particular sequences. The GeneBook^®^ databases are composed of over 150,000 microbial genomes and gene sequences representing over 1000 bacterial, 5000 viral, 250 protists and 1500 fungal species, as well as over 5500 antibiotic resistant and virulence associated genes. Each GeneBook^®^ database was screened and cleaned for host genome sequences including human, pig, and dog genomes, followed by validation by analyzing each host genome as a query in the cleaned databases. The web portal is hosted at AWS cloud and can be accessed at: https://app.cosmosid.com/login.

Metagenomic analysis is based on a proprietary high performance data-mining k-mer algorithm, implemented by C, as the core engine. The analysis algorithm has two separable comparators: a pre-computation phase for the reference database and a per-sample computation. The input to the pre-computation phase is a reference microbial genome or antibiotic resistance GeneBook^®^ database, and its output is phylogeny trees, together with sets of variable length k-mer fingerprints (biomarkers) that are uniquely identified with distinct nodes, creating braches and leaves of the tree. The reference GeneBook^®^ database constitutes both publicly available genomes or gene sequences such as NCBI- RefSeq/WGS/SRA/nr, PATRIC, M5NR, IMG, ENA, DDBJ, CARD, ResFinder, ARDB, ARG-ANNOT, mvirdb, VFDB, as well as a subset of genomes sequenced by CosmosID and its collaborators. The second per-sample computational phase searches the 100s of millions of short sequence reads or contigs from draft assembly against the fingerprint sets. The resulting statistics are analyzed to give fine-grain composition and relative abundance estimates. Next, edit distance-scoring techniques are used to compare a target genome or gene with the reference set. The algorithm provides similar functionality to BLAST. Classification precision is maintained through aggregation statistics. Enhanced detection specificity is achieved by running the comparators in sequence. In summary, the two part analysis consists of first finding reads in which there is an exact match with a k-mer uniquely identified with a GeneBook^®^ reference database, and then statistically scoring the entire read against the GeneBook reference to verify that the read is indeed uniquely identified with that reference. For each sample, the reads from a species are assigned to the strain with the highest aggregation statistics. Similarly, the community resistome, the collection of antibiotic resistance genes in the microbiome, was also identified using the CosmosID, Inc., bioinformatics software package to query the unassembled sequence reads against the CosmosID curated antibiotic resistance gene database in an analogous manner to bacterial species identification.

Analyses of the bacterial sequencing data included Shannon alpha diversity (Shannon, 1948), principal coordinate analysis (PCoA) performed using the Bray-Curtis distance measure, stacked bar graphs and heatmaps based on relative abundance of each microorganism (%) in each sample using the NMF R software package (Gaujoux and Seoighe, 2010). Resistome analysis was performed by identification of antibiotic-resistance genes based on percentage of gene coverage for each gene as a function of the gene-specific read frequency in each sample. Antibiotic resistance gene PCoA was analyzed using the Bray-Curtis distance measure based on gene frequency as described. Statistical analyses were performed using Microsoft Excel 2016. *T*-test analyses were two-tailed with unequal variance.

### Data Availability

Fecal DNA metagenomics sequencing data are available in Sequence Read Archive (SRA)^3^, Accession SRP093227.

## Results

### SYN-006 Did Not Affect Systemic ERT Levels

**FIGURE 1:**
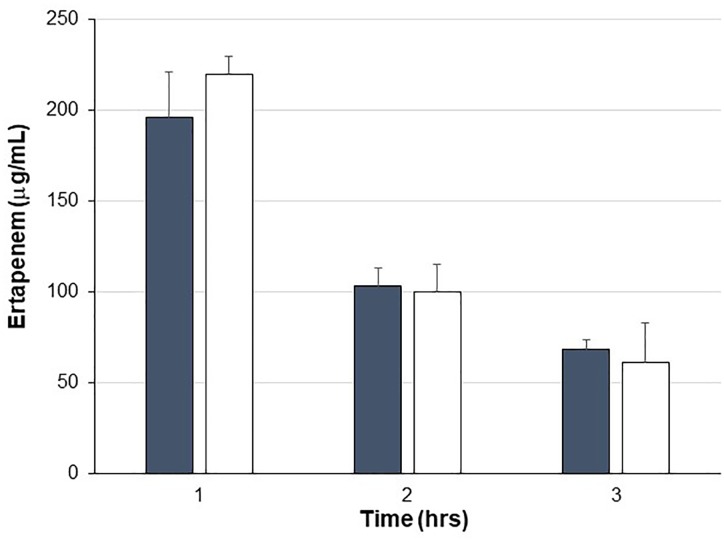
Ertapenem serum levels. Ertapenem (ERT) was measured in pig serum collected at the indicated times on day 3 of antibiotic treatment. Pigs were treated with ERT alone, gray bars or ERT + SYN-006, white bars. Data are displayed as mean + standard deviation. *P*-values were obtained by comparing ERT alone and ERT + SYN-006 groups at each time point using a two-tailed Student’s *t*-test. At 1 h, *p* = 0.42, 2 h, *p* = 0.86, and 3 h, *p* = 0.44.

Pigs were treated with IV ERT once daily for four consecutive days with one cohort also receiving SYN-006 delivered orally four times per day, starting the day before ERT treatment. To evaluate systemic antibiotic levels, blood was drawn on day 3 of antibiotic administration when animals had received three doses of ertapenem ±14 doses of SYN-006. ERT serum levels were not statistically different between ERT or ERT+SYN-006 cohorts at any time point (Figure 1), indicating that SYN-006 did not alter serum antibiotic levels in the pigs.

### SYN-006 Mitigated ERT-Mediated Microbiome Damage

**FIGURE 2:**
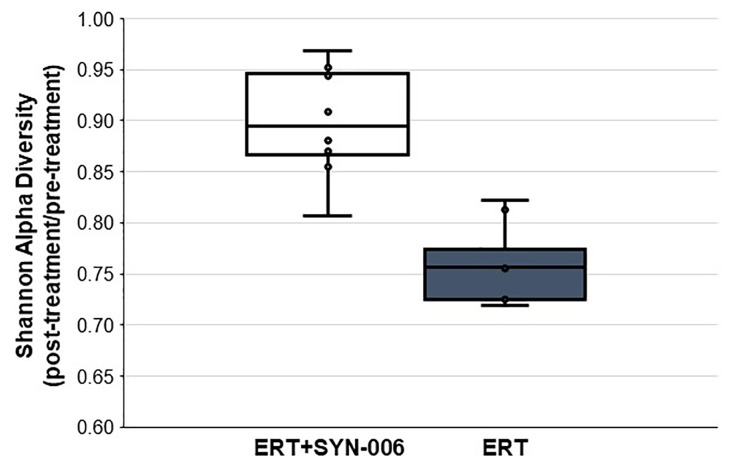
Shannon alpha diversity changes in the pig fecal microbiomes. Fecal microbiome metagenomics data were analyzed by Shannon alpha diversity and the post-treatment/pretreatment ratios were calculated for each animal. ERT alone, gray bar, ERT + SYN-006, white bar. *P*-values were obtained by comparing ERT and ERT + SYN-006 ratios using a two-tailed Student’s *t*-test, *p* = 0.00007.

**FIGURE 3:**
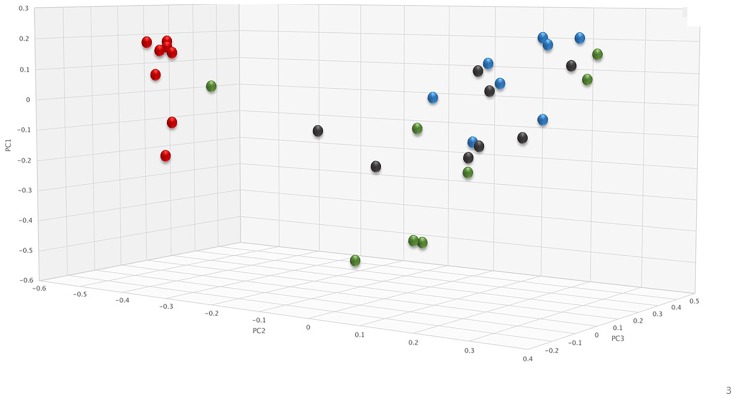
Principal coordinate analysis of fecal microbiomes. Relative abundance of bacterial species in the fecal microbiome of each animal at each time point were analyzed via principal coordinate analysis using the Bray-Curtis distance measure. Black: ERT pretreatment; red: ERT post-treatment; blue: ERT + SYN-006 pretreatment, and green: ERT + SYN-006 post-treatment.

To assess changes in the pig microbiomes, DNA isolated from feces collected before and after antibiotic treatment was subjected to whole genome shotgun metagenomic analyses. The relative abundance of the bacterial strains in each sample was determined to allow microbiome composition comparisons. Prior to ERT exposure, microbiomes displayed similar species richness with 472 ± 43 bacterial species identified in ERT microbiomes and 501 ± 27 species in ERT + SYN-006 microbiomes (*p* = 0.14). However, following antibiotic treatment bacteria species declined by >70% in ERT microbiomes (139 ± 35 species) and <50% in ERT + SYN-006 microbiomes (260 ± 57) demonstrating a significant reduction in bacterial richness between the two cohorts (*p* = 0.0003). Shannon alpha diversities were calculated and the change in microbiome species diversity after ERT exposure was compared for each cohort (Figure 2). ERT resulted in a significantly lower alpha diversity ratio compared to ERT + SYN-006 (*p* = 0.00007). Likewise, PCoA, using the Bray-Curtis dissimilarity index, compared post-treatment microbiome composition to pretreatment (Figure 3). Distance between points indicates degree of difference in sample composition with points close together more similar. Pretreatment samples clustered with ERT + SYN-006 post-treatment samples. In contrast, ERT post-treatment samples formed a distinct cluster. Notably, one ERT-SYN-006 post-treatment sample was closer to the ERT post-treatment samples than to the pre-treatment cluster, suggesting partial microbiome protection with SYN-006 in this animal (Pig 9).

**FIGURE 4:**
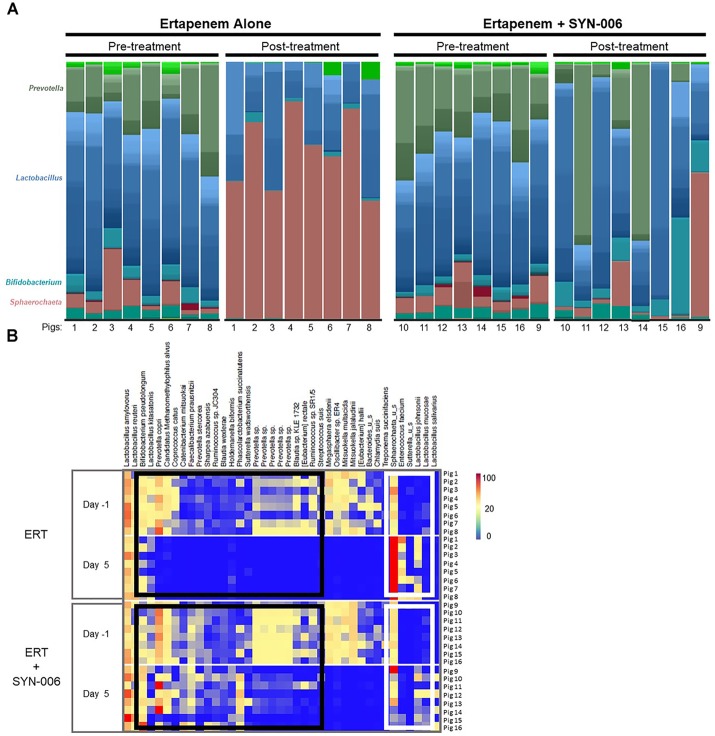
**(A)** Species level stacked bar graph of fecal microbiomes. Fecal microbiomes for each animal at each time point were analyzed via stacked bar graph comparing pre-treatment to post-treatment microbiomes. The genera of abundant species are displayed on the left, animal numbers are displayed on the bottom, and treatment groups and collection time point displayed at the top. **(B)** Heatmap analysis of the relative abundance of selected bacterial species present in the pig fecal microbiomes. Species composition of fecal microbiomes of pigs treated with ERT or ERT + SYN-006 are displayed as the abundance of each bacterial species relative to all species in each fecal sample. Each row represents an individual animal at the indicated time point. Bacterial taxa are indicated at the top of the figure, cohort and collection day are indicated on the left, and animal numbers indicated on the right. Black boxes display bacterial taxa diminished in the ERT cohort and maintained in the ERT + SYN-006 cohort. White boxes display bacterial taxa that proliferated with ERT and less so with ERT + SYN-006. The color gradient key displays a linear scale of relative abundance.

To visualize specific changes in the pig microbiomes, stacked bar graphs (Figure 4A) and heatmaps (Figure 4B) of the bacterial taxa were constructed, based on the relative abundance of bacterial species in each sample and organized to allow comparison of microbiomes before and after treatment. Compared to pretreatment microbiomes, ERT resulted in reduction and/or loss of specific bacterial species and overgrowth of other taxa, while ERT + SYN-006 microbiomes displayed fewer alterations in microbiota composition. ERT-exposed microbiomes showed a reduced abundance of commensals *Bifidobacterium pseudolongum, Prevotella copri, Coprococcus catus*, and *Faecalibacterium prausnitzii*, and overgrowth of *Sphaerochaeta* sp. and *Enterococcus faecium*, not observed in ERT + SYN-006 microbiomes (Figure 4B). Notably, *Sphaerochaeta sp.*, present at low abundance in all animals prior to antibiotic treatment, expanded to monodominance (47–85%) in all ERT microbiomes. In contrast, *Sphaerochaeta* monodominance was observed only in one post-treatment ERT + SYN-006 animal (56%, Pig 9). Similarly, *Prevotella copri*, present at high abundance in all animals prior to treatment, was not detected in the ERT animals, but was maintained in 6/8 ERT + SYN-006 animals post-treatment. Loss of several species, including *Megasphaera elsdenii, Oscillibacter sp., Mitsuokella multacida, Mitsuokella jalaludini*, and *Eubacterium hallii*, and overgrowth of *Lactobacillus johnsonii*, were observed in both ERT and ERT-SYN-006 microbiomes. These data demonstrate ERT caused major alterations in the gut microbiome composition that was attenuated by administration of SYN-006.

### SYN-006 Reduces Antibiotic Resistance Gene Propagation

**FIGURE 5:**
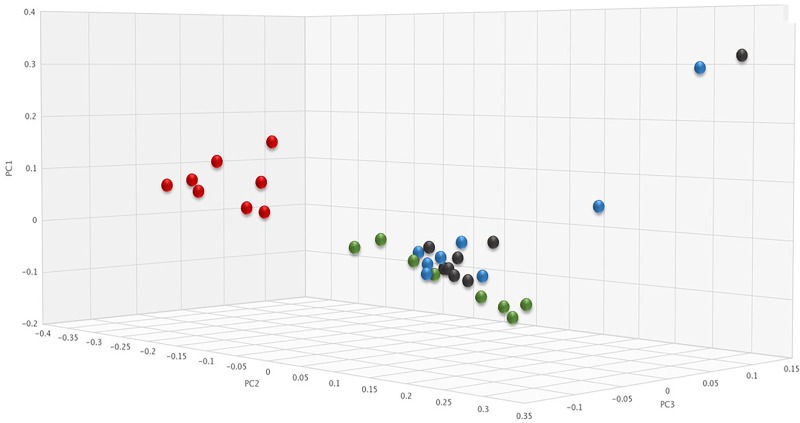
Principal coordinate analysis of fecal resistomes. Antibiotic resistance gene frequencies for each animal at each time point were analyzed via principal coordinate analysis using the Bray-Curtis distance measure. Black: ERT pretreatment; red: ERT post-treatment; blue: ERT + SYN-006 pretreatment, and green: ERT + SYN-006 post-treatment.

To determine if SYN-006 affected propagation of antibiotic resistance, the fecal DNA metagenomic data were analyzed for the presence of antibiotic resistance genes as a measure of the population of antibiotic resistant bacteria in the gut microbiome. PCoA analysis of antibiotic resistance gene frequencies, using Bray-Curtis dissimilarity, was performed to illustrate changes to the gut resistome (Figure 5). In general, pretreatment and ERT + SYN-006 post-treatment microbiomes clustered together while ERT microbiomes formed a distinct cluster. These data indicate ERT resistomes diverged more from pretreatment than the ERT + SYN-006 resistomes.

**FIGURE 6:**
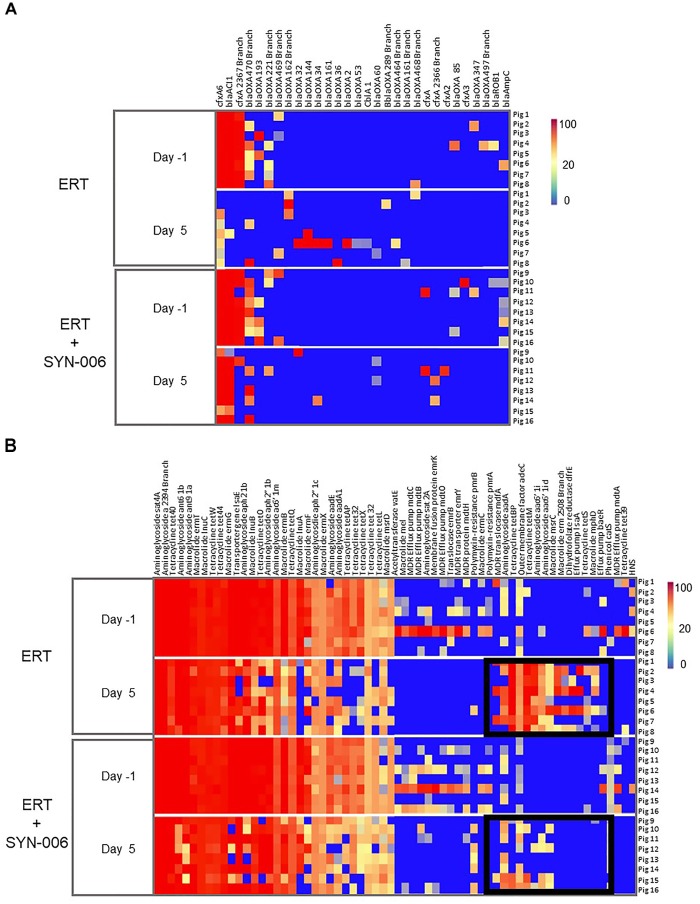
**(A)** Heatmap analysis of the frequency of beta-lactamase genes in the pig fecal microbiomes. Fecal microbiome metagenomics data were analyzed for the presence of antibiotic resistance genes based on the percentage gene coverage as a measure of relative gene frequency in each sample. Each row represents an individual animal at the indicated collection day. Beta-lactamase genes are identified at the top, treatment group and day of fecal collection on the left, and the animal numbers on the right. The color gradient key displays a linear scale of the percentage gene coverage as a measure of relative gene frequency. **(B)** Heatmap analysis of the frequency of antibiotic resistance genes in the pig fecal microbiomes. Fecal microbiome metagenomics data were analyzed for the presence of antibiotic resistance genes based on the percentage gene coverage as a measure of relative gene frequency in each sample. Each row represents an individual animal at the indicated collection day. Antibiotic resistance genes are identified at the top, treatment group and day of fecal collection on the left, and the animal numbers on the right. Black boxes display antibiotic resistance genes more prevalent in the ERT cohort compared to the ERT + SYN-006 cohort. The color gradient key displays a linear scale of the percentage gene coverage as a measure of relative gene frequency.

To visualize specific changes in resistomes, heatmaps of antibiotic resistance genes in the fecal microbiome of each animal, before and after antibiotic treatment, were generated (Figure 6). Several beta-lactamase genes, genes conferring resistance specifically to beta-lactam antibiotics, were observed following ERT exposure (Figure 6A) that included mainly those encoding Class D OXA beta-lactamases, many of which were carbapenemases (Antunes and Fisher, 2014; Antunes et al., 2014; Evans and Amyes, 2014). Of 12 *blaOXA* genes detected after ERT exposure, only three, *blaOXA 32, blaOXA 34*, and *blaOXA 60*, also were observed in ERT + SYN-006 resistomes. In contrast, *cfxA* and *cfxA2* genes were detected in three ERT + SYN-006 animals, but not in ERT-only resistomes. The *cfxA6* and *blaACl1* genes displayed reduced frequency following antibiotic exposure in ERT animals and were unaffected by ERT + SYN-006. Finally, *blaROB1* and *blaAmpC* genes were lost from both ERT and ERT + SYN-006 resistomes.

In addition to beta-lactamases, other resistance gene frequencies were altered by exposure to antibiotic (Figure 6B). Many of these genes encode components of multidrug efflux transporter systems that confer resistance to a broad range of antibiotics and others to specific antibiotic classes, such as aminoglycosides, macrolides, and tetracyclines (McArthur et al., 2013). In general, more genes were affected in ERT resistomes compared to those exposed to ERT + SYN-006. Increased frequency of components of multidrug efflux transporter systems (*mdfA, adeC, IsaA, baeR*), aminoglycoside (*aadA, aac6’1i*), macrolide *(msrC, erm, mphD*), and tetraclycine *(tetBP, tetM, tetS*) resistance genes were observed with ERT, which was attenuated in the presence of SYN-006. However, many resistance genes were lost with both ERT and ERT + SYN-006, including several multi-drug efflux pump components (*mdtO, emrY*), macrolide (*InuA, me*l), and tetracycline resistance genes (*tetX, tetL, tet32*) although reduction in frequency was more pronounced with ERT alone.

Taken together, microbiome and resistome analyses demonstrate that exposure of the porcine gut microbiome to the carbapenem antibiotic, ERT, caused rapid alterations in gut microbiome composition, resulting in emergence and propagation of selected populations of antibiotic resistance genes. ERT-mediated gut microbiome damage was attenuated by co-administration of SYN-006, an oral formulation of the novel metallo-beta-lactamase, P2A (Connelly et al., 2019).

## Discussion

Antibiotics negatively impact the gut microbiome and broad-spectrum beta-lactams are especially damaging (Falony et al., 2016; Ferrer et al., 2017). Of the beta-lactams, carbapenems are considered high risk for microbiome disruption and emergence of antibiotic resistance (Weiss et al., 2015; Connelly et al., 2018). Therefore, protection of the gut microbiome from all classes of beta-lactams, especially carbapenems, is anticipated to mitigate both short and long-term consequences of antibiotic-mediated gut dysbiosis, including emergence of carbapenem-resistant pathogens.

Here, carbapenem-meditated gut microbiome damage was mitigated by antibiotic inactivation in the GI tract following oral administration of SYN-006, in a large animal model. SYN-006 efficacy was evaluated using an established porcine model of carbapenem-mediated gut dysbiosis (Connelly et al., 2018) since the pig and human GI tracts share similar anatomy, immune function, and colonic microbiota composition (Rowan et al., 1994; Kararli, 1995; Xiao et al., 2016). Indeed, 96% of microbiota functional pathways found in the human microbiome are represented in pigs (Xiao et al., 2016). Pigs were used in a previous study evaluating ribaxamase efficacy of gut microbiome protection against IV ceftriaxone (Connelly et al., 2017). After 4 days of IV ERT treatment, pig gut microbiomes displayed significantly reduced alpha diversity (defined as the number of species and their relative abundance in each sample) compared to pretreatment. These data were confirmed by stacked bar graph and heatmap analyses demonstrating reduction or loss of some bacterial species and overgrowth of others. In contrast, with SYN-006, alpha diversity was significantly higher after antibiotic treatment compared to ERT alone, and fewer fluctuations in microbiota composition were observed. For example, commensals, including *Faecalibacterium prausnitzii* and *Coprococcus catus*, were undetectable after exposure to ERT, and *Enterococcus faecium*, not found prior to antibiotic exposure, emerged after ERT treatment. These species were comparatively unaffected with co-administration of SYN-006. Notably, changes in relative abundance of these taxa have physiological ramifications. Reduced levels of *F. prausnitzii* and *C. catus* have been associated with myalgic encephalomyelitis/chronic fatigue syndrome (Nagy-Szakal et al., 2017), while *E. faecium* has become recognized as the leading cause of multi-drug resistant enterococcal infection in the United States (Agudelo Higuita et al., 2014).

Of note was the dramatic overgrowth of an unclassified *Sphaerochaeta*, climbing to monodominance, defined as ≥ 30% relative abundance (Taur et al., 2012), in all animals in the ERT cohort but in only one ERT + SYN-006 animal, Pig 9. *Sphaerochaeta*, a recently described genus (Ritalahti et al., 2012), are members of the pig fecal microbial community (Pajarillo et al., 2014) and are inherently resistant to beta-lactam antibiotics (Caro-Quintero et al., 2012), thus explaining proliferation in the presence of ERT. In contrast, *Prevotella copri*, an abundant commensal susceptible to carbapenems (Shilnikova and Dmitrieva, 2015), was lost from all ERT microbiomes but only from two ERT + SYN-006 samples, including Pig 9. Notably, *P. copri* rose to monodominance in two ERT + SYN-006 microbiomes, demonstrating protection from ERT exposure. While these data indicate SYN-006 reduced ERT-mediated microbiome alteration, the results also suggest that SYN-006 protected the gut microbiome variably by animal, with Pig 9 representing partial protection. Pig 9 displayed overgrowth of *Sphaerochaeta*, combined with loss of *P. copri*, an observation similar to that of ERT microbiomes, while *F. prausnitzii* was maintained and *E. faecium* did not overgrow, similar to the pretreatment microbiomes. Consistent with the conclusion of incomplete protection is the observation that several species including *Megasphaera elsdenii, Mitsuokella multacida*, and an undefined *Oscillibacter* sp. were lost from both ERT and ERT + SYN-006 microbiomes.

To obtain more consistent microbiome protection, SYN-006 delivery can be improved. Most simply this is achieved by adjusting the dose to increase intestinal concentrations of SYN-006. Indeed, an *in vitro* bacterial growth assay, designed to model gut microbiota protection via antibiotic inactivation, showed complete beta-lactam degradation attained with increasing concentrations of SYN-006 (Connelly et al., 2019). Here, SYN-006 was delivered at a dose of 2.5 mg/kg (50 mg/20 kg pig, QID). In an analogous study using a pig model of ceftriaxone-mediated gut dysbiosis, effective microbiome protection was achieved by employing a ribaxamase dose of 3.75 mg/kg (75 mg/20 kg pig, QID) (Connelly et al., 2017), with much higher ribaxamase doses of 10 and 20 mg/kg/dose well tolerated in human and canine safety studies, respectively (Kokai-Kun et al., 2016; Roberts et al., 2016). SYN-006 did not affect ERT serum levels, suggesting it is not absorbed systemically, so delivery of 10-fold higher doses than used in the current study will likely improve microbiome protection without adverse systemic consequences.

Another approach that could be taken to potentially increase carbapenemase function would be to modify the SYN-006 formulation. The P2A carbapenemase is a class B1 metallo-beta-lactamase (Queenan and Bush, 2007) requiring zinc for antibiotic hydrolysis activity (Fabiane et al., 1998). For P2A enzyme production, biological activity was preserved by including zinc in all media and buffers (Connelly et al., 2019). To ensure improved enzyme function in the GI tract, additional zinc could be included in the SYN-006 formulation and/or patients could be advised to take a zinc supplement with each SYN-006 dose. In addition, at low pH (≤5.5) zinc ion binding is compromised resulting in loss of enzyme activity (Connelly et al., 2019). Therefore, proton pump inhibitors (PPIs) which raise the pH of intestinal fluid could be prescribed with SYN-006 during the course of antibiotic treatment. The use of PPIs is not expected to negatively impact enzyme function since PPIs had no effect on ribaxamase antibiotic degradation efficacy in a Phase 2a clinical study (Kokai-Kun J. F. et al., 2017).

In addition to microbiome damage, ERT exposure resulted in changes in the resistome that were attenuated in the presence of SYN-006. The observed resistome alterations were likely influenced by both direct selection of species harboring genes conferring resistance to ERT and by fluctuation in relative abundance of the gut microbiota following ERT exposure. For example, Class D OXA beta-lactamase genes that emerged following ERT exposure included *blaOXA-2, 32, 34, 36, 53, 144, 161*, and *162.* Notably, OXA-2 has recently been demonstrated to be a potent carbapenemase (Antunes et al., 2014), and OXA-32, 34, 36, and 53 are classified as OXA-2 like enzymes (Antunes and Fisher, 2014), suggesting that they also confer carbapenem resistance. Similarly, OXA-144 and OXA-162 are classified as OXA-48 and OXA-51 like enzymes, both known carbapenemases (Evans and Amyes, 2014). Therefore, it is not surprising that these genes appeared in ERT resistomes following antibiotic exposure. In contrast, Class A cfxA extended spectrum beta-lactamases (McArthur et al., 2013), observed after antibiotic administration in three ERT + SYN-006 animals, do not protect microorganisms from ERT. However, cfxA genes are present in *Prevotella* species (Giraud-Morin et al., 2003), and overgrowth of *P. copri* was observed in two animals exposed to SYN-006. Likewise, changes in species relative abundance may explain why some genes conferring resistance to aminoglycosides, macrolides, and tetracyclines emerged with ERT exposure while others encoding similar resistances were lost after antibiotic administration. Unfortunately, metagenomic sequencing data do not yet allow identification of bacterial taxa possessing specific resistance genes nor does it verify expression of these genes and therefore actual antibiotic resistance. However, these data are consistent with other reports demonstrating alterations in frequency of a broad range of antibiotic resistance genes after antibiotic exposure in pigs, and not just genes conferring resistance to the administered antimicrobial agent (Looft et al., 2012; Connelly et al., 2017, 2018). In a previous study, most beta-lactamase genes appeared in the pig resistome after 7 days of ERT administration, consisting of mainly *blaOXA* genes, while microbiome composition fluctuations occurred more rapidly, within 4 days of antibiotic exposure (Connelly et al., 2018). Here, the shorter, 4 days ERT regimen allowed evaluation of SYN-006 microbiome protection efficacy, but, may not have been optimal for observation of resistome changes. A follow-up study where animals are monitored for a longer time after antibiotic administration would provide valuable information regarding ERT-mediated consequences.

Protection of the gut microbiome from antibiotic-mediated damage is critical for maintenance of health. The widely used, broad-spectrum beta-lactams, including carbapenems, can be especially destructive. SYN-006, an enteric formulation of a novel carbapenemase, was demonstrated to mitigate gut microbiome disruption and reduce emergence of antimicrobial resistance caused by administration of a carbapenem antibiotic, ERT, in a pig model of antibiotic-mediated gut dysbiosis. SYN-006 has the potential to expand microbiome protection via antibiotic inactivation in the GI tract to all classes of beta-lactam antibiotics, and to attenuate emergence of carbapenem resistance.

## Author Contributions

SC and MK designed the experimental studies and interpreted the data. BF and NAH coordinated the sequencing of the fecal DNA and performed the sequencing data analyses. SC and BF produced the figures. BF submitted the fecal DNA metagenomics sequencing data to the SRA. SC wrote the manuscript. BF, NAH, RRC, and MK reviewed and edited the manuscript. All authors read and approved the final manuscript.

## Conflict of Interest Statement

The authors declare the following potential conflicts of interest with respect to the research, authorship and/or publication of this article: SC and MK are employees of Synthetic Biologics, Inc. RRC is the founder of CosmosID, Inc., a fee-for-service provider engaged by Synthetic Biologics, Inc. BF and NAH are employees of CosmosID, Inc.

## Footnotes

1 https://jgi.doe.gov/data-and-tools/

2 https://www.illumina.com/science/education/sequencing-quality-scores.html

3 https://submit.ncbi.nlm.nih.gov/subs/sra/
